# Climate change-induced migration: a bibliometric review

**DOI:** 10.1186/s12992-021-00722-3

**Published:** 2021-07-03

**Authors:** Juan Milán-García, José Luis Caparrós-Martínez, Nuria Rueda-López, Jaime de Pablo Valenciano

**Affiliations:** Department of Economics and Bussiness, University of Almeria, Almeria, Spain

**Keywords:** Climate change, Bibliometrics, Migration, Sustainability

## Abstract

**Background:**

This paper has reviewed the international research on the terms “climate change” and “human migration” from 1999 to 2019. To this end, a bibliometric and a cluster analysis by fractional accounting have been carried out using two of the most important databases: Web of Science (WoS) and Scopus. The research found and studied 140 documents from WoS Core Collection and 193 from Scopus.

**Results:**

The results show a continual increase in the number of articles published and citations received during the whole period studied. The U.S., U.K., Germany and China have been shown to be the most productive countries and there is a predominance of North American organizations supporting and fostering research on these topics.

**Conclusions:**

The main contribution of this article is the analysis of new tendencies. The trend shows a transition from concepts such as vulnerability, climate change, land degradation, refugees and security to others such as concepts such as international migration, climate justice, sustainability, human rights and disaster risk reduction. Future research in this field should address the comparison of results from research focused on human beings to a focus on other living beings.

## Introduction

Throughout the history of the planet, climate has undergone major changes which can be clearly verified in the geological record. However, since the Industrial Revolution, when fossil energy consumption increased exponentially and, therefore, the emission of greenhouse gases, this process of climate change has been more a consequence of human action than of natural phenomena.

For some scientists, such is the transformation and magnitude of the changes induced in the environment that they have formulated the idea that we live in a new geological epoch, characterised by the power of humans as a force of transformation on a global scale: the Anthropocene [7, 15].

At present there is broad scientific consensus [14], practically universal, that our current model of production and energy consumption is responsible for the planet-wide climatic alteration, unprecedented in the history of mankind. While it is currently having a serious impact on both the environment and the world economy, everything indicates that it will be exacerbated in the future.

To this end, since its inception in 1988, the Intergovernmental Panel on Climate Change (IPCC) has been informing about the causes and possible repercussions of this phenomenon, as well as proposing response strategies.

The IPCC’s fifth report, in which more than 830 scientists from 85 countries participated, concluded that “warming in the climate system is unequivocal and human influence is clear”. Some of the indisputable evidence of this climate change, which the aforementioned report includes, is the increase of 0.85 °C in the average temperature of the Earth since the beginning of the twentieth century, the increase of 0.2 m. of the average sea level [27].

On the other hand, increasingly prolonged and severe droughts worldwide as well as extreme weather events such as devastating fires, torrential rains or hurricanes, are becoming more frequent and intense [50].

The impact of this phenomenon is manifesting itself virulently in some areas of the planet causing changes in the way of life of millions of people. Climate Change is destroying crops, killing herds or making traditional lifestyles unfeasible in entire regions of the planet.

Such is its impact, that there are studies that state that Climate Change is currently considered to be a more important driver of migration than economic and political factors in the countries of origin [34, 62].

Related to this, the latest report by the World Bank Group [53] concludes that if urgent measures are not taken in the fight against Climate Change and Development, by 2050, the number of displaced persons as a result of this phenomenon could reach 140 million in three of the most densely populated developing regions in the world (South of the Sahara, South Asia and Latin America). The people of these lands would be forced to leave their lands as a result of water scarcity, poor harvests, rising sea levels and tsunamis.

On the other hand, recent studies have shown that Climate Change has been one of the contributing factors for the unleashing of armed conflicts such as the recent one in Syria [13, 52]. Climate change caused this country to suffer a major drought during the period 2007 to 2010, leading to a series of crop failures and the consequent displacement of millions of people from rural areas to cities, resulting in overcrowding, unemployment and major political unrest that eventually led to civil war [1, 11].

However, the evolution of the concept of Climate Change has undergone changes over time [49]. Together with the deterministic conceptions that accept the reality of climate change, discrepant and critical voices have arisen that call into question its existence or even reject the concept altogether (Table 1).

**Table 1:** Definitions of climate change

Author	Definition
Todorov, A.V. (1986) [60]	The concept of climate change is both complex and controversial. There is no unanimous opinion and agreement among climatologists on the definition of the term climate, not to mention climate change, the trend or climatic fluctuation.
United Nations (Bodansky, 1993) [9]	A variation in the climate attributed directly or indirectly to human activity that alters the composition of the world’s atmosphere and that adds to the natural variability of the climate observed in comparable periods of time.
Lorenz, E. (1995) [Lorenz EN: Climate is what you expect. Unpublished]	Climate is the current distribution of a climate system over time that extends indefinitely into the future, so there is no talk of the existence of climate change.
IPCC (Parry et al., 2007) [46]	A change in the state of climate that can be identified (for example, by statistical tests) by changes in the average and / or the variability of its properties, and that persists for a prolonged period, usually of decades or more.
Werndl, C. (2014)	Different climatic distributions in two successive periods of time.

The above notwithstanding, the increase in evidence that refutes the existence of the phenomenon proves that Climate Change, especially in developing countries, can amplify existing vulnerabilities and instabilities in these regions and cause a marked increase in the number of displaced people. According to the forecasts made by the scientific community, the influence of this phenomenon on migration can lead to risks to public safety and health [55, 57].

Migratory movements as a consequence of environmental causes have been studied in the past. However, it has only been in recent decades that the international scientific and political community has taken a special interest in identifying and assessing the link between both variables, even to the extent that the International Organization for Migration [43] has proposed a definition for people who are forced to migrate due to climate related changesenvironmental consequences.

So called “environmental migrants” are people or groups of people who, due to a sudden or progressive change in the environment that adversely affects their lives, are forced to leave their habitual homes, either temporarily or permanently, and who move either within their country or abroad [30].

The interest that this issue has raised in the scientific community is such that in the last decade the number of articles which link Climate Change to an increase in migratory movements that are a result of attempts to adapt to the environment [8, 20, 23, 29, 35, 36, 38, 64]. Of note are studies carried out in Africa [22, 25, 39, 42, 58], Asia [12, 17, 40, 56, 59, 65], America [21, 37, 41, 54, 63] and, to a lesser extent, Europe [16, 28].

For this reason, we believe that it is very useful for the scientific community in this area of study to know the evolution of the publications that relate these two concepts, migrations and climate change, as well as the main areas of knowledge in which they have been developed.

The aim of this bibliometric study is to analyse the evolution of the scientific literature related to these two concepts (migration and climate change), not only to find out the current state but also where research trends in this field are heading to.

The knowledge generated by this study can be a very useful tool for the study, design and implementation of new research on the subject, as well as for the formulation of policies aimed at reducing the vulnerability of populations exposed to environmental risk factors that can lead to migration.

## Materials and methodology

The method used to analyze the concept of migration in the context of climate change is a bibliometric analysis. This is a scientific method widely accepted by leading research institutions such as the National Science Foundation or the European Commission [51], which uses statistical and mathematical techniques to evaluate research results [48] at various levels (by countries, authors, magazines, research centers, among others) taking as a reference the principle of the citation network [45]. In addition, the h-index is used to explain the performance or production of research work. This is defined as the number of articles with a total number of citations ≥h [26]..

Articles, books, conference proceedings and other research documents have been included in the initial search, yielding a total of 439 documents in the WoS database and 408 in Scopus. However, these results have been filtered in the impact analysis to only include articles (Table 2). The reason for this is due to the fact that these types of documents adhere to a strict review process that ensures the quality of their content, particularly with regards to the results and conclusions. Finally, information related to migration and climate change has also been filtered by encoding the material obtained and analyzing it.

**Table 2:** Distribution of publications by document type

	WoS	Scopus
Article	321	263
Book and Book Chapter	65	102
Review	14	30
Proceeding Paper	39	13

**Fig. 1:**
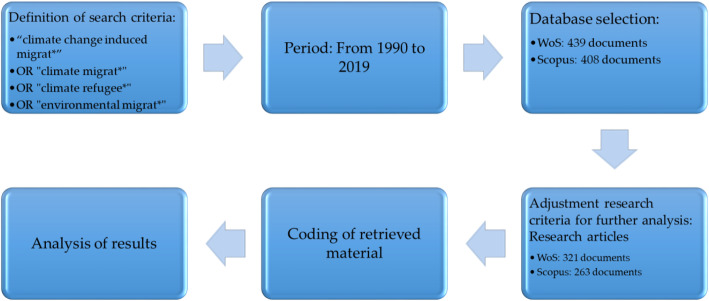
Keyword selection process

Using a fractional counting method, VOSviewer software has been used to perform cluster analyzes with which to build and visualize the bibliometric network [61]. This method states that each action, such as co-authoring or citing a population, has the same weighting regardless of the number of authors, citations or references of a publication [47]. The bibliometric analysis followed the following steps (Fig. 1). It commenced by defining the search criteria, the keywords and the study period. At first, it was decided to use the terms “climate change induced migrat*” OR “climate migrat*” OR “climate refugee*” OR “environmental migrat*” to analyze the impact of climate change on the migratory movements of people.

The study period coincides with the first article registered in each database until the year 2019 (Fig. 1). Once this was clarified, Scopus and WoS were the databases selected to perform the analysis, since they are the two most relevant data sources due to the rigorous protocol that they follow to ensure that the articles they include present a high level of quality [44].

The published work that most closely resembles this research is that of [33] entitled Environmental and climate migrations: an overview of scientific literature using a bibliometric analysis, in which they apply the bibliometric approach to the same issue. However, the main contributions of this research, unlike the one mentioned above, are to be found in a broader study period that includes the year 2019 as a whole, a document search that includes the concept of climate change induced migrations, as well as the comparative analysis of the WoS and Scopus databases.

## Results

### Number of publications per year

The first registered research on the impact of climate change on migration is found at the end of the 1990s in both the WoS and Scopus database with the work by Fang and Liu [18] entitled “*Relationship between climatic-change and the nomadic southward migrations in eastern Asia during historical times*” in which the basis for historical research on climate change-induced migratory movements are set from the analysis of nomadic migrations in South Mongolia and East Asia (See Table 3).

**Table 3:** Annual distribution of publications

	WOS				SCOPUS			
Y	A	TC	TC/A	H	A	TC	TC/A	H
1992	1	47	47	1	1	62	62	1
1993	2	4	2	1	2	8	4	2
1994	1	16	16	1	2	19	9.5	1
1996	1	30	30	1	3	38	12.7	1
1997	3	118	39.3	3	3	138	46	3
1998	–	–	–	–	1	8	8	1
2004	1	16	16	1	–	–	–	–
2006	1	32	32	1	1	34	34	1
2007	2	23	11.5	2	3	400	133	2
2008	2	92	46	2	5	171	34.2	4
2009	3	170	56.7	3	6	184	30.7	3
2010	14	733	52.4	9	16	818	51.1	10
2011	13	508	39.1	10	10	518	51.8	8
2012	15	356	23.7	7	13	382	29.4	7
2013	20	453	22.7	10	21	488	23.2	11
2014	34	126	3.71	7	16	142	8.88	8
2015	37	415	11.2	13	33	397	12	13
2016	34	135	3.97	6	24	140	5.83	6
2017	38	134	3.53	7	23	82	3.57	5
2018	54	142	2.63	7	32	129	4.03	7
2019	41	23	0.56	3	37	25	0.68	3

*Y* Year, *A* Articles, *TC* Total Cites, *H* h-index

**Fig. 2:**
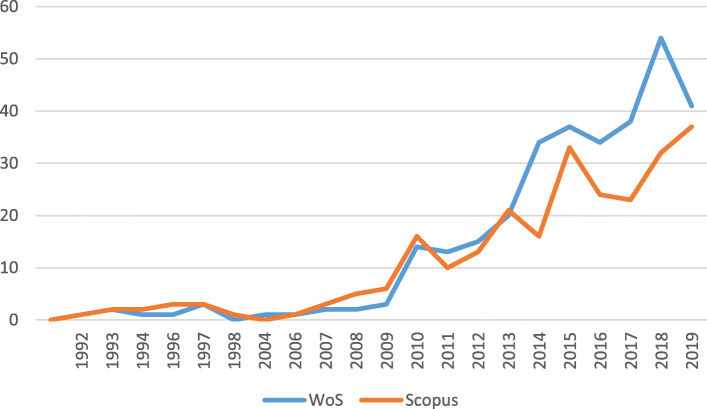
Evolution in the number of articles

Since those first publications, the number of articles published has steadily and regularly increased since 2009, with WoS including a greater number of articles than Scopus throughout much of the period (Fig. 2). The maximum point is reached in 2015 for Scopus, coinciding with the rejection of the United States to the Kyoto Protocol to combat climate change, and 2018 for WoS. This is evidence that the impact of climate change on migration is a matter of rigorous relevance in the scientific community.

**Fig. 3:**
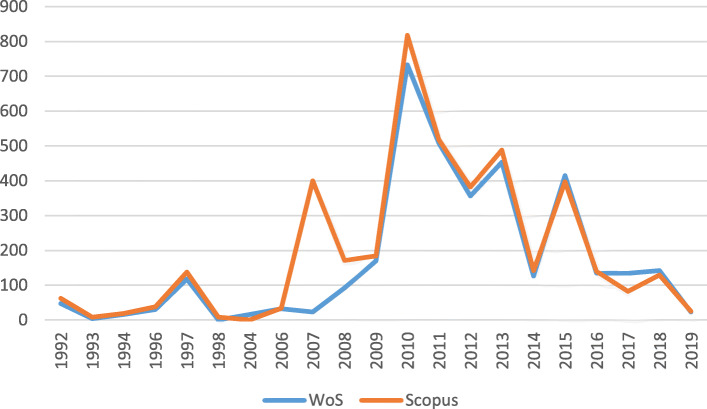
Evolution in the number of citations

On the other hand, the evolution in the number of citations presents a more irregular trend to the one shown in relation to the number of articles. As shown in Fig. 3, these attain their highest level in 2010. However, the most striking data is found in 2007 in Scopus: with only 3 articles registered, the total number of citations rises to 400. This is due to Reuveny’s work entitled *Climate change-induced migration and violent conflict* (371 citations) in which the conflicts that arise with the arrival of climate migrants in new territories are addressed. In fact, it is the most widely quoted article on Scopus. In contrast, the WoS database contains [19] research entitled *The first climate refugees? Contesting global narratives of climate change in Tuvalu* (177 citations) which addresses the concept of climate refugees.

### Distribution by knowledge area

The distribution by knowledge area shows a concentration on issues related to environmental and social sciences (See Table 4).

**Table 4:** Distribution by knowledge area

Research areas WOS	Articles	Research areas scopus	Articles
Environmental Sciences	173	Social Sciences	178
Geography	48	Environmental Science	174
Law	40	Art and Humanities	27
Demography	35	Economics	18
International Relations	26	Energy	17
Political Science	21	Engineering	5

### Distribution by institution

The institution with the largest number of articles published, according to WoS, is the University of Minnesota, followed by the University of London and University of Colorado. Similarly, on Scopus the ranking are led by the University of Minnesota and the University of Wollongong along with the University of Colorado (Table 5). The majority of the most influential institutions are in the United States, followed by the United Kingdom, China and Australia.

**Table 5:** Distribution by institution

Institution	Country	Articles		Total cites		TC/A		H-index	
		W	S	W	S	W	S	W	S
University of Minnesota	USA	12	10	129	90	11	9	7	5
University of London	UK	11	2	44	5	4	2.5	3	1
University of Colorado	USA	9	6	182	172	20	28.67	4	4
Lancaster University	UK	7	4	33	12	4.7	3	3	2
Macquarie University	Australia	7	3	23	6	3.3	2	2	1
University of Wollongong	Australia	7	8	436	466	62	58.25	5	5
Chinese Academy of Sciences	China	3	5	43	63	14	12.6	2	4

*W* WoS, *S* Scopus

The University of Minnesota is ranked 30th in the world ranking of universities in 2014, due to its outstanding economics program. The University of Colorado, for its part, has had among its professors noble awards in the field of physics and chemistry, such as John L. Hall or Herbert Kroemer. The University of London is one of the oldest institutions in the country, standing out in the fields of economics and management, medicine, mathematics and law. Finally, the University of Wollongong stands out in Information and Communication Technologies, Engineering and Geography, among others.

### Distribution by author

The distribution by authors shows that the largest number of articles is published, in WoS, by both databases, is Mayer, while on Scopus there is a tie between Bettini, Hunter, Riosmena and Boas, but it is the latter one who has the highest number in total citations. All authors began their research career in this branch of knowledge in the twenty-first century (Table 6). Mayer’s most cited article is titled *““Environmental migration” as advocacy: is it going to work?”* (17 citations), which addresses the governance of environmental migration. Boas, on the other hand, in the article, *“Preparing for a warmer world: Towards a global governance system to protect climate refugees”* (194 citations), which addresses a global governance project for the protection and voluntary resettlement of climate refugees.

**Table 6:** Distribution by author

Authors	ID	Articles		Total cites		TC/A.		H-index		1st article	Last article
		W	S	W	S	W	S	W	S		
Mayer, B.	55347613800	13	3	42	22	3.23	7.33	4	2	2011	2018
Nawrotzki, R.J.	55326263700	8	4	83	59	10.4	14.8	5	3	2014	2018
Bettini, G.	55445709900	7	5	146	130	20.9	26	4	3	2013	2019
Hunter, L.M.	7202851675	6	5	160	154	26.7	30.8	3	3	2010	2018
Farbotko, C.	14017584600	5	4	325	339	65	84.8	4	4	2010	2019
Gemenne, F.	36863235700	5	2	15	136	3	68	2	2	2015	2018
Riosmena, F.	23486359000	5	5	57	46	11.4	9.2	3	3	2015	2018
Boas, I.	26038946900	4	5	252	286	63	57.2	3	3	2008	2019

*W* WoS, *S* Scopus

### Distribution by journal

The distribution of articles by journal shows that *Global Environment Change*, *Population and environment* and *Regional Enviornmental Change* are the most influential in both databases (Table 7). This coincides with the thematic distribution presented above, in which the knowledge areas of environmental science is the most relevant in the field of migration and climate change.

**Table 7:** Distribution by journal

Source title	Impact factor	Articles		Total cites		TC/A.		H-index	
		W	S	W	S	W	S	W	S
Global Environment Change	10.427 (Q1 JCR)	12	13	622	772	51.8	59.4	12	13
Population and Environment	0.67 (Q1 Scopus)	11	12	64	89	5.82	7.42	6	5
Regional Environmental Change	3.149 (Q2 JCR)	6	6	91	101	15.2	16.8	4	4
Sustainability	2.592 (Q2 JCR)	5	5	17	18	3.4	3.6	3	2
Global Environment	0.19 (Q2 Scopus)	5	4	5	4	1	1	2	1
Climatic Change	4.168 (Q1 JCR)	4	4	57	73	14.3	18.3	2	3
Environmental Research Letters	6.192 (Q1 JCR)	4	4	62	59	15.5	14.8	3	3
Environmental Science and Policy	4.816 (Q1 JCR)	4	4	111	125	27.8	31.3	3	2
Geoforum	2.926 (Q1 JCR)	4	4	234	248	58.5	62	3	3
Journal of International Development	0.74 (Q1 Scopus)	4	4	199	228	49.8	57	4	4

*W* WoS, *S* Scopus

### Distribution by country and language

The distribution by countries shows that the United States, United Kingdom, Australia and Germany are the most relevant countries in both WoS and Scopus (Table 8), followed by Canada and China. This coincides with the results obtained in the distribution by institutions.

**Table 8:** Distribution by country

Country	WOS				Country	SCOPUS			
	A	TC	TC/A	H		A	TC	TC/A	H
USA	82	1229	15	17	**USA**	68	1666	24.5	18
UK	45	506	11.2	11	**UK**	36	518	14.39	11
Australia	39	750	19.2	14	**Australia**	26	619	23.80	11
Germany	39	418	10.7	11	**Germany**	26	299	11.5	9
Canada	23	239	10.4	6	**Canada**	19	276	14.53	6
China	18	147	8.17	5	**China**	13	168	12.92	6
France	12	150	12.5	4	**France**	9	264	29.33	5
Switzerland	12	141	11.8	5	**Austria**	9	95	10.56	6
Italy	11	5	0.45	1	**India**	9	55	6.11	4

The distribution by language supports the results obtained from the distribution by institutions and countries, that is, English, Chinese, German, French and Spanish (Table 9) are the most used languages in articles relating to the impact of climate change on migration.

**Table 9:** Distribution by language

Language	Articles	
	WoS	Scopus
English	294	246
Spanish	15	3
French	3	5
Chinese	1	5
German	–	2

### Recent keyword trends

**Fig. 4:**
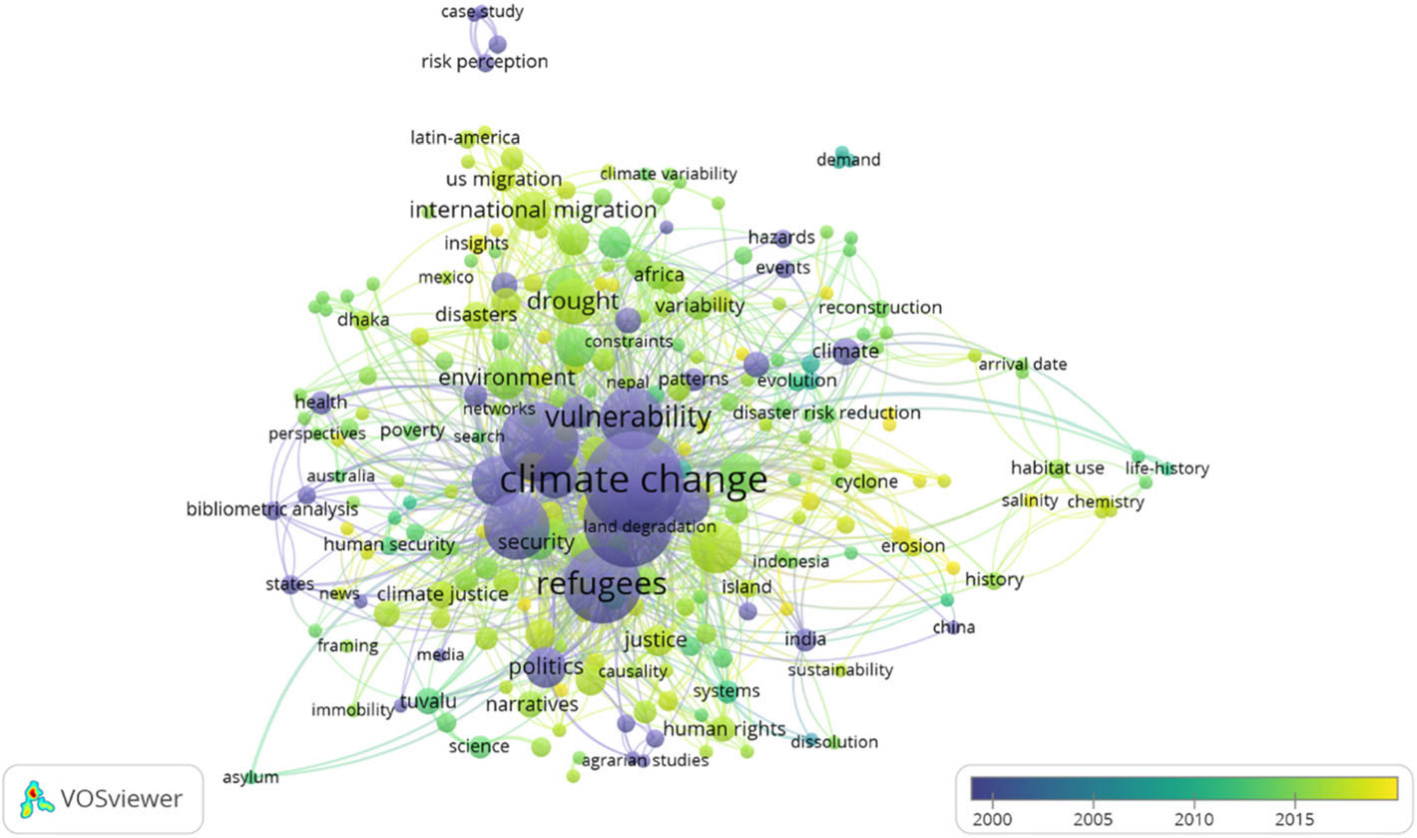
Analysis of New Trends

For the analysis of new trends a fractional counting cluster of the keywords of the entire period has been carried out, and includes keywords that have been used in at least two or more articles. The different configuration of the clusters of new trends can be seen in Fig. 4.

The color scale indicates the most current keywords: from a violet color that represents keywords from 2000 to a yellow color that represents keywords of papers published after 2015. Specifically of note is the fact that at the beginning of the period, the most utilized keywords were vulnerability, climate change, land degradation, refugees and security. By the end of the study period, concepts such as international migration, climate justice, sustainability, human rights and disaster risk reduction appear to be more prevalent.

## Discussion

In recent years, scientific interest in the causes and effects of the phenomenon of climate change has increased [31], and related branches of research have developed, as shown in Fig. 4. In relation to the migratory effect caused by climate change, since 2016 there has been an increase in the number of articles that use the term climate justice, defined as the imperative need to include the environmental dimension in all decision-making (regardless of the corresponding discipline), with a legal framework that allows action to be taken in the event that this dimension is not taken into consideration [6]. In the scientific literature, there are several works that relate climate justice to climate change-induced migration, such as the research by Ajibade et al. [3], who emphasise the importance of differentiating climate migration from managed retreat to enhance climate justice, or the study by Ahmed [2] focused on identifying the responsibility of climate refugees.

As shown in Fig. 4, other strands in the last 5 years relate climate change-induced migration movements to sustainability [4, 24], human rights [5, 10] and disaster risk reduction [32].

Overall, recent trends show that the phenomenon of climate change induced migrations is a warning of the ruinous consequences of climate change consolidation across the globe. It is therefore essential for all citizens to make a commitment to the environment in order to guarantee the sustainability of future generations.

## Conclusions

In this work, the international research trends followed by researchers in the context of human migration and climate change from the end of the twentieth century until 2019 have been analyzed. For this, a bibliometric analysis has been carried out with articles from the theWoS and Scopus databases, with a final sample of 321 articles from WoS and 263 from Scopus. The results indicate that the number of articles published per year has uniformly increased throughout the period, particularly from 2010 until its peak in 2018. This shows that international scandals such as the rejection by the President of United States of the existence of climate change and non-compliance with the Kyoto Protocol aroused the interest of the research community. This is confirmed by the evolution in the number of citations which, despite having a more irregular growth compared to the number of articles published, also reaches its highest points in 2007, 2010 and 2015. In light of the above, it follows that research on human migration in the context of climate change has been shown to be a growing area of study in recent years in the WoS database, while on Scopus, interest has declined since 2015.

*Global Environment Change*, *Population and environment* and *Regional Enviornmental Change* are the journals with the largest number of publications throughout the period. The United States is the country with the largest number of published articles, followed by the United Kingdom, Australia and Germany. This distribution by countries is reflected in the distribution by institutions, with the University of Colorado and Minnesota (United States), the Univerisity of Wollongong (Australia) and the University of London (United Kingdom) have the largest number of articles published in both databases, while the most utiltized languages in this field of research are English, Chinese, Spanish, French and German.

With regards to the distribution of authors on WoS, Mayer, Nawrotzki, and Bettini have the largest number of articles published. On Scopus, Hunter, Boas, Riosmena and Bettini have the largest number. This notwithstanding, Farbotko is the author with the highest number of citations per article, together with Boas and Hunter, who publish articles on topics related to environmental sciences, social sciences and geography.

The keyword trend analysis shows a convergence in terms of the concepts of climate change and human migration in recent years. Throughout the study period, there is a transition from concepts such as vulnerability, climate change, land degradation, refugees and security to others such as concepts such as international migration, climate justice, sustainability, human rights and disaster risk reduction. This shows that the scientific community is more focused on the consequences of climate change not only on the state of health of the population, but also in the patterns of national and international migratory movements for the purpose of seeking work and in the decision-making processes of planning and organizing local territories.

On the limitations of this research it should be noted, first, that the field of study has been restricted only to the most influential academic databases (WOS and Scopus). Second, only articles have been analyzed. Therefore, it would be interesting to broaden the research to include other databases such as Google Scholar and other types of publications such as books or conference proceedings.

Due to the high impact that climate change has not only on people’s lives, but also on global biodiversity as a whole, it would be interesting to compare the results of the current research on both aspects, identifying differences and similarities that allow us to know if as humans, we display the same behavior as other species facing changes in the environment.

## Data Availability

The datasets during and/or analysed during the current study available from the corresponding author on reasonable request.
